# DBS in tremor with dystonia: VIM, GPi or both? A review of the literature and considerations from a single-center experience

**DOI:** 10.1007/s00415-023-11569-6

**Published:** 2023-01-21

**Authors:** Davide Paoli, Russell Mills, Una Brechany, Nicola Pavese, Claire Nicholson

**Affiliations:** 1 Clinical Ageing Research Unit, Newcastle University, Campus for Ageing and Vitality, Newcastle upon Tyne, NE4 5PL UK; 2 Present Address: Neurology Unit, Department of Clinical and Experimental Medicine, University of Pisa, Ospedale Santa Chiara, 56126 Pisa, Italy; 3 Newcastle upon Tyne Hospitals NHS Foundation Trust, Newcastle upon Tyne, NE1 4LP UK; 4 Newcastle Magnetic Resonance Centre and Positron Emission Tomography Centre - Newcastle University, Campus for Ageing and Vitality, Newcastle upon Tyne, NE4 5PL UK; 5 Department of Nuclear Medicine and PET Centre, Aarhus University Hospital, Aarhus, Denmark

**Keywords:** Dystonia, Tremor, Deep brain stimulation, Thalamus, Globus pallidum

## Abstract

**Background:**

Deep brain stimulation (DBS) is an established treatment for dystonia and tremor. However, there is no consensus about the best surgical targeting strategy in patients with concomitant tremor and dystonia. Both the thalamic ventral intermediate nucleus (VIM) and the globus pallidus pars interna (GPi) have been proposed as targets. Few cases using them together in a double-target approach have also been reported.

**Methods:**

We reviewed the literature on this topic, summarizing results of different target choices. Additionally, we retrospectively report a case series of nine patients with sporadic dystonia and severe tremor treated with a double-target strategy at our center. Outcome measures were the Burke-Fahn-Marsden Dystonia Rating Scale (BFM) and Eq-5d scale.

**Results:**

In published studies of patients with dystonia and tremor, VIM-DBS is highly effective on tremor but raise some concerns about dystonia’s control, while GPi-DBS is more effective on dystonia but does not always relieve tremor. GPi + VIM-DBS shows good efficacy but is rarely reported and reserved for selected patients. In our patients, the double-target strategy obtained a significant and durable improvement in tremor, dystonia, and quality of life. Additionally, compared with a cohort of patients with tremor treated with VIM-DBS only, significantly lower frequency and intensity of VIM stimulation were required to control tremor.

**Conclusion:**

Our findings and published evidence seem to support the double-targeting approach as a safe and effective option in selected patients with tremor-dominant dystonia. This strategy appears to provide a more extensive control of either dystonia or tremor and may have a potential for limiting stimulation-related side effects.

**Supplementary Information:**

The online version contains supplementary material available at 10.1007/s00415-023-11569-6.

## Introduction

Dystonia is defined as a “movement disorder characterized by sustained or intermittent muscle contractions causing abnormal, often repetitive, movements, postures or both”. This definition includes many different and heterogeneous manifestations which are classified according to their characteristics and etiology [1].

Nowadays, tremor is universally accepted as one of the possible manifestations of dystonia, though its severity and its relationship with dystonic features and their distribution can vary considerably between patients. As a result, there is considerable variation in the extent to which symptoms affect a patient’s quality of life and cause disability [2].

Differentiating between tremor in dystonic syndromes and other tremor disorders, particularly essential tremor (ET), remains challenging due to the overlap between these two diseases and the lack of reliable diagnostic tools. It is well known that ET can show mild dystonic features (ET plus) and tremor may sometimes be the dominant characteristic of dystonic patients, with only mild, sometimes intermittent, dystonia. However, asymmetry, coarseness, and irregularity of shaking are clinical clues suggestive of a dystonic etiology and there is growing evidence of the potential value of neurophysiological studies in confirming the diagnosis [2, 3].

In the last two decades, surgery has been offered to treatment-resistant dystonic patients with associated tremor when the condition leads to significant disability or discomfort. Both deep brain stimulation (DBS) and ablative approaches have been demonstrated to be feasible and effective, with the former being generally preferred due to reversibility and the possibility of modulating the stimulation parameters. However, the choice of the best DBS target for these patients remains an open question, with a current debate between the thalamic ventral intermediate nucleus (VIM) and the globus pallidus pars interna (GPi) [4–6]. A combined approach, targeting both GPi and VIM has also been proposed [5, 6]. This approach has proved to be feasible and was suggested as a good option in patients with more severe impairment, but evidence in this field is still limited and only a small number of cases have been reported [6–10].

The aim of this article is to review the literature about this topic and to retrospectively discuss a case series of nine patients with tremor-dominant dystonia implanted with bilateral GPi + VIM electrodes between 2014 and 2019 at our site.

## Methods

### Review of published literature

A PubMed search was performed of all literature published from 1st January 1990 using the following terms: (Dystonia OR Tremor OR Dystonic tremor) AND (surgery OR deep brain stimulation OR thalam* OR pallid* OR therapy OR treatment). Papers included in this review met the following criteria: (I) written in English; (II) focused on human subjects with tremor and dystonia or dystonic tremor undergoing surgical treatment for their condition; (III) provided a clinical description of cases and a clear quantitative or qualitative outcome of the procedure. When an article met the inclusion criteria, its references were searched for further papers. Exclusion criteria were: (I) editorials, comments, notes or letters without any data and/or recommendations; (II) studies with aims inconsistent with the scope of the review (e.g., studies investigating dystonic patients without tremor; (III) articles without peer review or in which peer-review process was still pending (defined as “preprint”); (IV) studies not including human subjects.

Cases from different articles were then grouped depending on the three most common diagnoses [sporadic segmental or multifocal dystonia; primary writing tremor (PWT); other forms of either acquired or inherited dystonia with associated hyperkinetic disorders]. In each diagnosis subgroup, cases treated with VIM stimulation, GPi stimulation, or double-target VIM and GPi stimulation were distinguished. Articles describing DBS targets other than the GPi or VIM, and ablative surgery rather than DBS were also considered.

### Our center experience

Nine consecutive patients with severe tremor and dystonia, not responsive to medical therapy, received dual-target DBS in our center at the Royal Victoria Infirmary, Newcastle Upon Tyne Hospitals NHS Trust (NUTH), UK between September 2014 and October 2018.

Seven of those patients had never undergone any surgical procedure for their neurological disorder and underwent bilateral implantation of both GPi and VIM leads in the same surgical session. One further patient underwent bilateral implantation of both GPi and VIM electrodes after removal of bilateral VIM leads, previously implanted in another center. Finally, our last patient had VIM surgery only since she had already had implanted GPi leads in 2012 with unsatisfactory control of her tremor.

All patients had complex clinical features, with coexisting moderate-to-severe tremor and mild-to-moderate dystonia. They were all diagnosed with “adult onset, segmental or multifocal dystonia, progressive and persistent, combined with tremor” and tremor was the most disabling symptom in all patients. Classification on Axis 2 of the new classification of dystonia [1] was “sporadic”.

Four patients had a family history of tremor and/or dystonia. Three of them (pt5, pt8, and pt9) underwent genetic testing that ruled out a known genetic etiology for their disease, including the most common forms associated with tremor [DYT-TOR1A (DYT1); DYT-THAP1 (DYT6); DYT-GNAL (DYT25); DYT-ANO3 (DYT-24)] [11].

#### Pre-surgical assessment

Before surgery, each patient underwent the standard of care pre-DBS assessment at our center, including a careful motor assessment with clinical rating scales, a video recording, and a thorough neuropsychological evaluation to identify any possible contraindication to the procedure, particularly cognitive or mood issues. All patients but the two with a previously implanted DBS system, had a brain MRI performed before the procedure.

Each case was discussed in a multidisciplinary team meeting to establish their suitability for DBS surgery.

#### Retrospective assessment of tremor at baseline

Severity of tremor at baseline and its distribution were retrospectively assessed by rating the videotapes taken before surgery. Tremor was mainly present in the head and upper limbs. However, legs tremor or voice tremor was not always rateable. Consequently, we decided to systematically rate only head and upper limbs (rest, postural, and action) tremor. For all patients, we assessed items 4.1 (head rest tremor), 5 (right upper limb: rest, postural, and intention tremor), and 6 (left upper limb: rest, postural, and intention tremor) of the Fahn-Tolosa-Marin Rating Scale. The maximum possible score (worst score) in this subscale is 28 (0–4 for each item, 7 sub-items).

Based on the distribution of tremor and its spatial relationship with the localization of dystonic signs, individual patients were retrospectively classified as having “dystonic tremor” or “tremor associated with dystonia”, according to current criteria [2].

#### Activation of the leads, programming, and follow-up

There are no reports available in literature about a formal protocol to activate four DBS leads in patients with sporadic dystonia and tremor. Our main objective was to obtain the best possible outcome for each patient rather than validate a specific stimulation protocol. Therefore, the four DBS leads were activated in a patient-tailored fashion trying to obtain a clinically relevant benefit in the shortest possible time and employing both pallidal and thalamic electrodes, unless side effects occurred. During their follow-up, patients were reviewed on an individual basis with the aim of maintaining or improving symptoms control. If symptoms worsened, or if side effects occurred, patients underwent extra clinical reviews. In some patients, reprogramming, including activating or deactivating some of the leads or contacts, was needed.

#### Outcome measures

Outcome measures were retrospectively collected from the assessments of our centre’s dystonia protocol, which includes a baseline evaluation and mandatory review evaluations at 6, 12, and 24 months after surgery. At these visits, each patient was assessed by the Burke-Fahn-Marsden Dystonia Rating Scale (BFM); the extent to which symptoms affecting quality of life were evaluated with the EuroQol scale for Health-related quality of life scale (Eq-5d) [12, 13]. Changes in these scales were employed as a primary outcome measure.

For all other reviews, clinical changes/side effects and full neurological examination were recorded in the clinical notes and letters to the patients General Practitioners, but no rating scales were performed.

Finally, we compared parameters of VIM stimulation in these patients at last available appointment with those obtained from a mixed cohort of patients with ET or dystonic tremor from our center who had received VIM-DBS only (control cohort). Details about the methodology and statistical analysis are provided in Supplementary Materials in the paragraph “Supplementary analysis—Methods”.

All data in the manuscript and tables are expressed as mean ± standard deviation.

## Results

### Review of literature

Four hundred and seventy-eight articles were found on Pubmed. All of them were screened, and only those fulfilling the criteria reported in the Methods section were included. Their references were subsequently screened to identify further articles, as previously discussed. Overall, we found 27 papers on surgical treatment of different forms of tremor in patients with dystonia; results are detailed in Tables 1 and 2. Settings, when available, are detailed in Supplementary Table 1. It must be specified that we only reported the final outcomes and the final settings in these papers. Furthermore, some articles included patients with dystonia and tremor of different etiologies; therefore, they may be cited in different sections of this paragraph.

**Table 1:** Synopsis of papers included in the review targeting VIM, GPi or both in patients with different forms of tremor and dystonia

Ref	Author	Year	Patients (n)	Surgical strategy and target	Tremor: improvement	Dystonia: improvement	Notes or clinical details
			(1) Sporadic, adult-onset dystonia				
[4]	Cury	2017	6	VLp DBS (4 UL, 2 BL) *One patient had previously received UL thalamotomy*	41%	30% (but deterioration over time)	-Three patients were successively implanted with GPi due to the worsening of dystonic symptoms
[6]	Hedera	2013	4	VIM-DBS (3 UL, 1 BL)	93%	− 10%	
[7]	Morishita	2010	2	VIM-DBS (1 UL, 1 BL)	40%%	NA	
[10]	Woehrle	2009	1	VIM-DBS (BL)	Marked	58%	
[14]	Vercueil	2001	3	-Right VLp DBS on previous Left thalamotomy (*n* = 2) -Bilateral VLp DBS (*n* = 1)	Moderate to marked	Not satisfactory	Bilateral DBS was performed in a case of head dystonic tremor
[16]	Buhmann	2013	1	Ventral-lateral thalamic base DBS (BL)	60%	71.40%	Cervical dystonia with head dystonic tremor
[19]	Deuschl	2002	1	VIM-DBS (BL)	Successful control	Mild progression	
[6]	Hedera	2013	4	Gpi-DBS (BL)	47	63	
[14]	Vercueil	2001	1	GPi-DBS (BL) (*previous VLp but progression of dystonia*)	Moderate	68%	Chronic stimulation with GPi only
[15]	Torres	2010	1	Gpi-DBS (UL)	75	60	Cervical dystonia with head dystonic tremor
[17]	Valalik	2011	1	Pallidotomy (UL)	Suppressed	88%	Meige syndrome with head dystonic tremor
[18]	Krause	2004	1	Gpi (BL)	Unsatisfactory	17% (transient)	Cervical dystonia with head dystonic tremor
[9]	Schadt	2007	1	Double target DBS (BL VIM + BL GPi)	Marked	Marked/dramatic	
[6]	Hedera	2013	2	Double target DBS (UL VIM + BL GPi)	55%	64%	One patient had received previous contralateral thalamotomy
[7]	Morishita	2010	1	Double target DBS (BL VIM + BL GPi)	45%	70%	
			(2) Primary writing tremor				
[20]	Lyons	2013	1	VIM-DBS (UL)	100%	Na	
[21]	Racette	2000	1	VIM-DBS (UL)	90%	Na	
[22]	Minguez Castellanos	1999	1	VIM-DBS (UL)	85.20%	Na	
[23]	Meng	2018	1	VIM-Vop MRI-FUSS (UL)	Suppressed	Na	
			(3) Other forms				
[10]	Woehrle	2009	1	VIM BL DBS	Marked improvement	59.50%	
[24]	Loher	2009	1	Vop UL Thalamotomy	Slight improvement	Slight improvement (marked on torticollis)	Post-traumatic pontomesencephalic lesion
[25]	Alvarez	2014	1	VIM UL thalamotomy	Suppressed	Suppressed	Post-Thalamic stroke
[26]	Carvalho	2014	1	GPi UL DBS	80%	Not reported	Post-traumatic (thalamic lesion)
[8]	Oropilla	2010	1	Double target UL GPi + UL VIM-DBS	62%	80.77%	Myoclonic dystonia
[27]	Kuncel	2009	1	VIM BL DBS	100%	Not reported	Myoclonic dystonia
[28]	Coenen	2018	1	GPi BL DBS	Suppressed	Very marked improvement (tongue)	Mohr–Tranebjaerg syndrome

Some articles report more than one target or different forms of dystonia; therefore, the same article may appear more than once in the tables

*BL* bilateral, *DBS* deep brain stimulation, *GPi* globus pallidus pars interna, *MRI-FUSS* magnetic resonance imaging focussed ultrasound stereotactic surgery, *UL* unilateral, *VLP* ventrolateral posterior nucleus, *VIM* ventral intermediate nucleus, *Vop* ventral oralis posterior nucleus

**Table 2:** Synopsis of papers included in the review targeting structures other than GPi or VIM in patients with different forms of tremor and dystonia

Ref	Author	Year	Patients (n)	Surgical strategy and target	Tremor: improvement	Dystonia: improvement	Notes or clinical details
[29]	Pauls	2014	7	Subthalamic DBS (BL)	57%	70%	Cervical dystonia with head dystonic tremor A trial thalamic vs subthalamic leads was performed
[16]	Buhmann	2013	2	Ventral thalamic base and/or PSA DBS (BL)	64.60%	73.80%	Cervical dystonia with head dystonic tremor
[30]	Blomstedt	2009	2	PSA DBS (UL)	100%	Not reported	Hand dystonic tremor (sporadic and acquired, post-traumatic, respectively)
[33]	Kitagawa	2000	1	Subthalamic area DBS (UL)	Remarkably reduced	Reduced	Upper Limb idiopathic dystonic tremor
[32]	Jeong	2009	1	Subthalamic area DBS (BL)	64.9%	80.50%	Acquired (lesional) dystonia and tremor
[31]	Chou	2005	1	STN DBS (BL)	suppressed	very marked reduction	Limbs and cervical dystonic tremor in sporadic dystonia
[34]	Plaha	2008	1	cZi DBS (BL)	70.50%	65%	Limbs dystonic tremor in sporadic dystonia

*BL* bilateral, *cZi* caudal zona incerta, *DBS* deep brain stimulation, *STN* subthalamic nucleus, *PSA* posterior subthalamic area, *UL* unilateral

#### Sporadic dystonia (either focal, segmental or multifocal) with tremor

Eleven papers [4, 6, 7, 9, 10, 14–19] reported patients with sporadic dystonia (either focal, segmental or multifocal) with tremor among the symptoms. In total, they reported 30 patients, 18 treated with thalamic stimulation or ablation only, 8 with pallidal surgery, and 4 with simultaneous stimulation of GPi and VIM.

Thalamic surgery [4, 6, 7, 10, 14, 16, 19] resulted in a considerable improvement in tremor (moderate to marked, 58.47% when quantitative outcomes were provided) in all papers. The benefit on dystonia was less consistent among the included articles. Some authors described a worsening of dystonia [6, 14, 19], while two reports, respectively, stimulating the inferior thalamic base [16] and the VIM [10], reported a good benefit on dystonic symptoms too. Time from thalamic surgery and the onset of the benefit was uniformly reported to be brief. However, over time, a progressive loss of benefit was described by some authors [4, 14]. For this reason, in one study [4], three of the six patients originally treated with VIM-DBS were successively implanted with GPi leads; no further information about successive stimulation settings or outcome in these patients was provided.

GPi-DBS led to a 63% improvement in dystonia and a 47% improvement of tremor in a series of four patients with segmental or multifocal disease[6]. In focal cervical dystonia with dystonic head tremor, two papers reported, respectively, 75% and 100% improvement in tremor and 41% and 90% improvement in dystonia following unilateral GPi-DBS or unilateral pallidotomy, respectively [15, 17]. However, another group reported no benefit from bilateral GPi-DBS in a patient with a severe dystonic cervical tremor [18].

Finally, we found one report of chronic pallidal stimulation [14] in a patient who had previously been treated with DBS of the ventrolateral thalamic nucleus (VLp), with significant improvement of tremor but generalization of dystonia. In this patient, after GPi-DBS activation and deactivation of the VLp leads, the benefit on tremor was retained and a consistent amelioration of dystonia was also achieved.

Multitarget DBS, addressing both GPi and VIM, was performed in four cases of sporadic dystonia combined with tremor [6, 7, 9]; three patients had received two-stage surgery, with VIM leads added after an unsatisfactory response to GPi stimulation, and one patient a one-stage double-targeting implantation. Overall, with this strategy a benefit reported as “marked” was achieved both on dystonic and tremor symptoms: only one case reported less than 50% improvement of tremor, while the minimum improvement of dystonia was 64%.

One additional paper [10] reported four further patients with sporadic dystonia and marked dystonic tremor who were implanted with both GPi and VIM electrodes. However, in these patients, the leads were externalized for test stimulation for several days to screen for the best clinical effect. Eventually, only one target was selected for chronic stimulation: three received chronic pallidal stimulation and one patient received chronic Vim DBS.

#### Tremor in dystonic syndromes

Eleven articles reported outcomes of surgery in eleven cases with tremor in other dystonic syndromes. Four cases were affected by PWT [20–23], four had acquired dystonia with tremor due to brain ischemic or traumatic lesion [10, 24–26], two were cases of tremor in patients diagnosed with myoclonic dystonia [8, 27], and one was a case of Mohr–Tranebjaerg syndrome [28].

All the four PWT cases had received thalamic surgery [three thalamic DBS, one magnetic resonance-guided focussed ultrasound surgery (MRgFUS)]. Improvement was reported to range between 85 and 100%.

Of the four cases [10, 24–26] with acquired dystonia, two were treated with thalamotomy [24, 25], one with GPi-DBS [26] and one with VIM-DBS [10]. All these cases reported a good benefit on both dystonic symptoms and tremor.

One of the two patients with significant disabling tremor in the context of a myoclonic dystonia was treated with both GPi and VIM-DBS [8], while the other received VIM stimulation only [27]. Both patients experienced significant improvement of all features considered (myoclonus, dystonia, and tremor) [8, 27].

Finally, the patient with Mohr–Tranebjaerg syndrome responded well to pallidal DBS [28].

#### Surgical targets other than the GPi and thalamus

Seven papers [16, 29–34] described surgical targets other than the GPi and thalamus. Their description is beyond the aim of this review. However, their target and outcome are detailed in Table 2.

### Our center case series

#### Demographics and baseline characteristics

Data regarding gender, age, disease duration, and BMF scores at baseline for the nine patients are summarized in Table 3. Briefly, the mean total BMF score was 34.44 ± 9.08; the mean motor sub-score was 23 ± 7.02; the mean disability sub-score was 11.44 ± 2.96; and the mean Eq-5d score was 23.89 ± 7.41.

**Table 3:** Baseline assessments of the nine patients included in the study

	Gender	Age	Disease duration	BMF—total	BMF—motor	BMF—disability	Eq-5D
Pt 1	F	45	26	24	14	10	20
Pt 2	F	67	14	44	32	12	30
Pt 3	M	58	8	46	31	15	25
Pt 4	M	49	35	42	28	14	15
Pt 5	M	70	40	31	20	11	20
Pt 6^a^	M	64^a^	12^a^	32^a^	18^a^	14^a^	20^a^
Pt 7	F	75	4	40	29	11	20
Pt 8	F	68	37	20	15	5	40
Pt 9^b^	F	48^b^	18^b^	31^b^	20^b^	11^b^	25^b^
Mean		***60.44***	***27.5***	***34.44***	***23.00***	***11.44***	***23.89***
SD		***10.88***	***13.44***	***9.08***	***7.02***	***2.96***	***7.41***

Mean values and Standard Deviation (SD) are highlighted in bold, italic

^a^Patient underwent GPi + VIM implant after removal of previously implanted VIM leads

^b^Patient underwent VIM implant but had a pre-existing GPi, active since 2012

The average score for tremor, assessed as described above, was 12.00 ± 4.2. Tremor severity and distribution are reported in Table 4.

**Table 4:** Scores for retrospective assessment of tremor at baseline of patients included in the study, obtained according to the method described in the “Methods” section

	Head	Right upper limb			Left upper limb			Total
		Rest	Postural	Action	Rest	Postural	Action	
*Pt 1*	2	0	0	2	0	0	2	6
*Pt 2*	2	1	3	4	1	3	4	18
*Pt 3*	1	2	2	2	1	2	2	12
*Pt 4*	2	0	3	4	2	4	4	19
*Pt 5*	1	2	2	3	0	1	2	11
*Pt 6*	2	2	2	2	1	2	1	12
*Pt 7*	0	0	0	3	2	2	3	10
*Pt 8*	2	1	2	1	1	1	0	8
*Pt 9*	2	1	3	3	1	2	2	14

On a diagnostic perspective, six patients (pts 1, 2, 3, 5, 8, 9) met criteria for “dystonic tremor”. The other three patients (pts 4, 6, and 7) had tremor far beyond localization of their dystonic features, consequently meeting the criteria for “tremor associated with dystonia”. In particular, they all had head and bilateral upper limbs tremor, but pt4 and pt6 only exhibited asymmetric limb dystonia and pt7 had neck dystonia only.

#### Follow-up

Mean follow-up duration was 30.56 ± 14.65 months. Outcome measures were available for all patients at 6 and 12 months. The 24-month assessment was available for seven patients. In fact, one case (pt9) had only recently completed the 12 months of follow-up, and another patient (pt6) had moved to another DBS center after the first 12 months because of the distance from our center.

#### Outcome

After 6 months, the mean total BMF score was 12.00 ± 5.05 with an average improvement of 64.2% compared to the baseline score. The motor sub-score improved to 6.00 ± 4.64 with a mean reduction of 73.1% The disability sub-score was 6.00 ± 1.73 with an average improvement of 44.4%. Eq-5d score rose to an average of 64.22 ± 15.75 (168% increase compared to the baseline value).

One year after surgery, the mean total BMF score was 10.78 ± 4.29; overall, the improvement was 67.8%. The motor sub-score was 5.44 ± 4.10 with a mean reduction of 75.8% compared to baseline. The disability mean sub-score was 5.33 ± 1.66, with an average improvement of 49.9%. Eq-5d assessment resulted in an average score of 68.89 ± 12.41 (188% increase compared to the baseline value).

After 2 years, for the seven patients who completed this follow-up, the mean total BMF score was 10.14 ± 3.34, with an average improvement of 68.8% compared to baseline; the motor sub-score was 4.86 ± 1.46, with a mean reduction of 77.1%; the average disability sub-score was 5.29 ± 2.36, with an average improvement of 47.6%. Eq-5d assessment resulted in a mean of 70.29 ± 11.66 (194% increase compared to the baseline value).

Tremor was markedly improved in all patients throughout the observational period, contributing to the improvement in Eq-5d score and the reduction of the BMF disability sub-score. The only exception was pt8, who showed an unsatisfactory control of tremor with a mild improvement only.

**Fig. 1:**
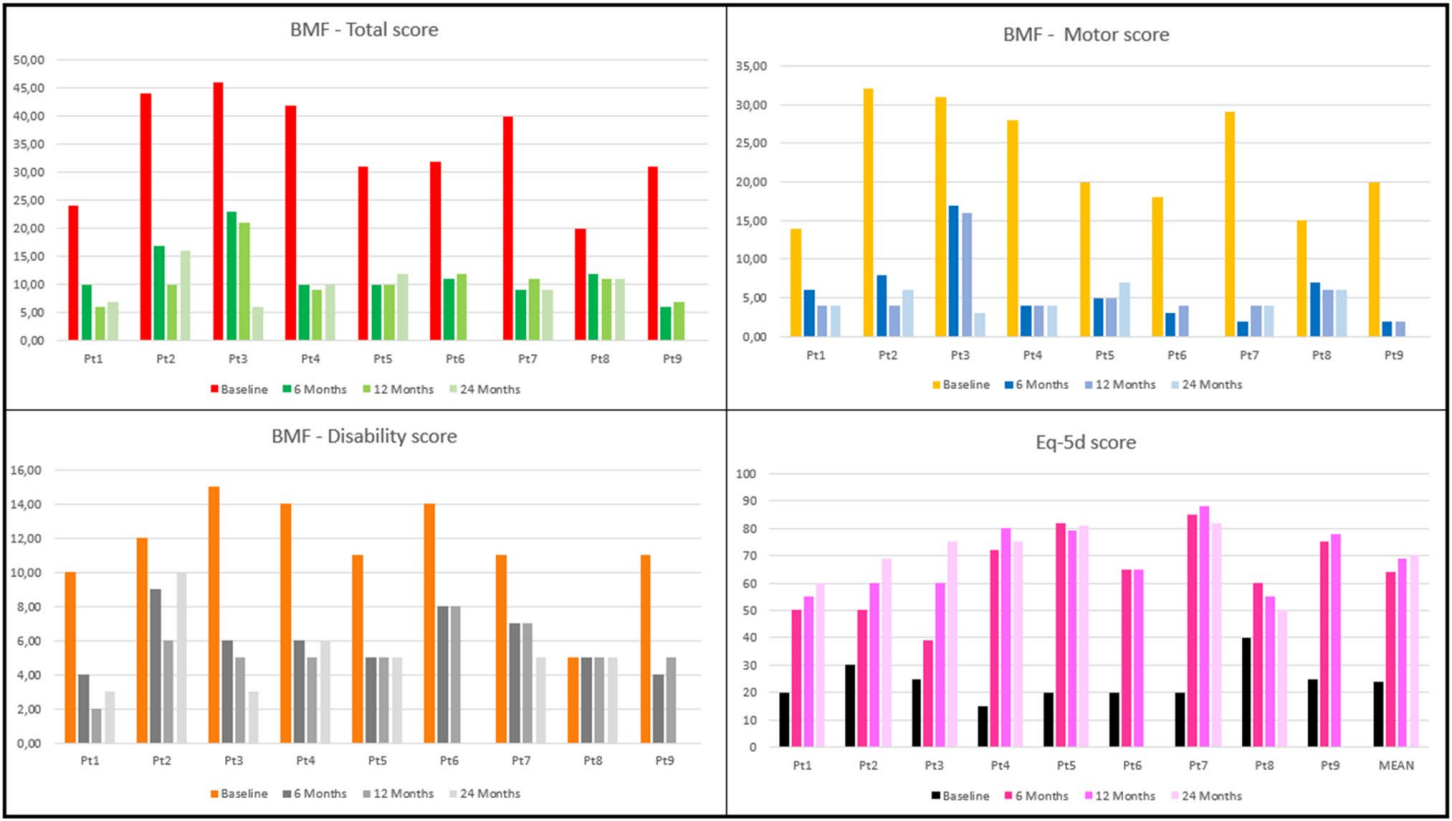
Graphic representation of the improvement of the Burke-Fahn-Marsden Dystonia Rating Scale (BMF) [total score (top, left); motor score (top, right); disability score (bottom, left)] and Eq-5d score (bottom, right) in individual patients during the follow-up period, as assessed at 6, 12, and 24 months (24 months not available for pts 6 and 9)

**Fig. 2:**
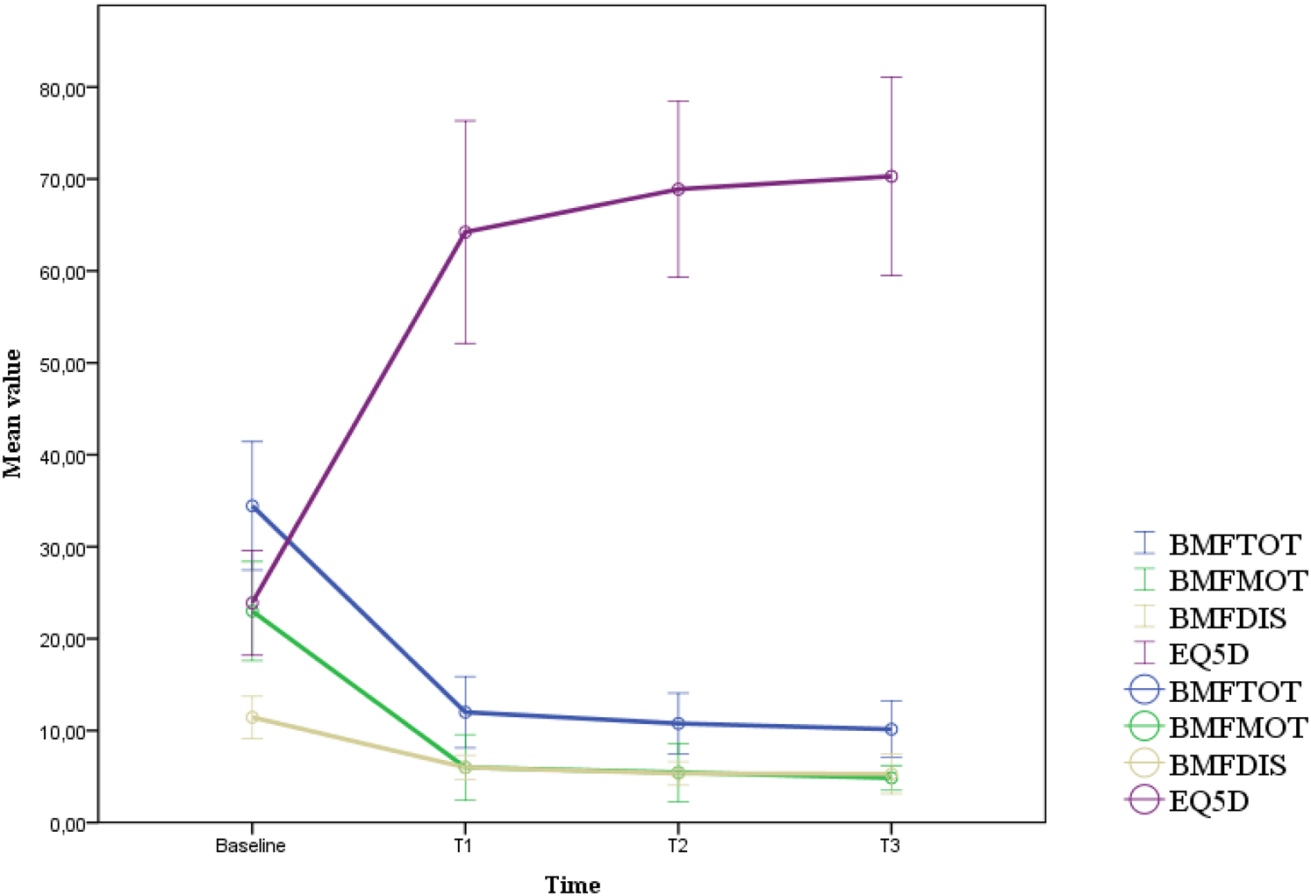
Graphic representation of the mean improvement of the Burke-Fahn-Marsden Dystonia Rating Scale [total score; motor score; disability score] and Eq-5d score

A graph of the improvement of every patient is provided in Fig. 1, while a representation of the mean improvement is summarized in Fig. 2.

#### Side effects and complications

After surgery, two patients experienced a slower than expected recovery, particularly related to mood and cognitive function. One of them also experienced mild hemiparesis of the right upper limb, though there was no evidence of intracerebral hemorrhage or other abnormality on post-operative imaging. He completely recovered after 2 months.

We documented reversible side effects possibly related to stimulation in five out of nine patients. The most common was slurred speech (5/5), followed by balance problems (4/5), and fluctuations in mood and concentration (1/5).

After careful reprogramming of DBS settings, speech problems improved in three patients and balance issues in two. In the remaining patients, there was no improvement of these symptoms not even after temporary switching DBS off, suggesting that they might not be related to the stimulation.

One patient needed further surgery for revision and then substitution of the IPG battery in the second year after surgery.

#### Major changes in stimulating configurations

After activating the DBS system, all patients had all their electrodes activated. As previously described, each patient could ask for a review in case of side effects or a worsening of their symptoms.

In two patients (pt4, pt6), revising and reprogramming were required after 9 and 8 months from surgery, respectively, due to the onset of side effects, as previously discussed, together with a not completely satisfactory control of tremor. Both patients were switched from a double targeting configuration to VIM stimulation only. Following deactivation of the GPi electrodes, there was a very mild worsening of the dystonic features, as the average BMF total score increased from 10 to 10.5 and the mean value of motor sub-score increased from 3 to 4, while the disability sub-score remained unchanged.

Pt7 had GPi leads turned off for a period of 11 months as he was complaining of mild side effects. However, motor symptoms control was unsatisfactory and consequently all the four leads were reactivated and were on at the time of the last assessment.

In pt8, right VIM stimulation caused slurred speech, leading to switching off the right thalamic lead.

Stimulation settings of patients at their last available appointment are reported in Table 5.

**Table 5:** Summary of the stimulation settings of patients included in the study, at their last available appointment

	Device	Left VIM	Right VIM	Left GPi	Right GPi
Pt1	Boston scientific	3 + ,2- 1.0 mA 40 msc 119 Hz	6-,7 + , 1.0 mA 40 msc 119 Hz	case + ,10- 1.2 mA 120 msc 119 Hz	14-,case + 1.2 mA, 120 msc, 119 Hz
Pt2	Medtronic Activa RC	case + ,2- 2.6 V 60 msc 125 Hz	case = 6-,5- 2.0 V 60 msc 125 Hz	case + ,10-, 1.0 V, 210 msc, 125 Hz	case + ,15- 1.2 V 210 msc 125 Hz
Pt 3	Boston scientific	case + , 2- 1.3 mA 60 msc 119 Hz	case + ,6- 1.4 mA 60 msc 119 Hz	case + 10- 1.0 mA, 210 msc, 119 Hz	case + ,13- 1.0 mA 210 msc 119 Hz
Pt 4	Boston scientific	case–,6-,5- 4.3 mA 40 msc 185 Hz	case + ,14-,13- 4.5 mA 50 msc 185 Hz	–	–
Pt 5	Boston scientific	case + , 1- 5.0 mA 120 msc, 185 Hz	8 + ,5- 4.7 mA 90 msc 185 Hz	–	Case + , 13-, 2.0 mA 90 msc 185 Hz
Pt 6	Boston scientific	case + ,1-,2-,3- 4.5 mA 60 msc 180 Hz	case + ,5-,6- 2.5 mA 60 msc 180 Hz	–	–
Pt 7	Boston scientific	case + ,2- 1.6 mA 40 msc 119 Hz	case + ,6- 2.9 mA, 60 msc 119 Hz	case + 10- 2.5 mA 210 mcs 119 Hz	case + 14- 1.8 mA 210 msc 119 Hz
Pt 8	Boston scientific	–	8 + ,5- 1.5 mA 60 msc 119 Hz	11-,10,case + 2.7 mA 40 msc 119 Hz	case + ,14- 3.8 mA, 120 msc, 119 Hz
Pt 9	Medtronic Activa RC	12-,case + 3.1 V 60 msc 125 Hz	case + ,9- 2.6 V 60 msc 125 Hz	2 + ,1–0- 2.4 V 120 msc 125 Hz	6 + ,5-,4 + , 2.2 V, 120 msc, 125 Hz

The comparison between parameters of VIM stimulation at last available appointment in the patients with four activated leads and those of the control cohort showed that current intensity was significantly higher in the latter (*p* value = 0.048). The same happened for frequency (*p* value = 0.041). No significant difference was found when comparing pulse width values. Full results of this analysis are described in the Supplementary Materials.

## Discussion

### Review of the literature

DBS is nowadays considered a gold standard treatment for severe and medical refractory tremor and dystonia, conventional targets are, respectively, VIM for tremor, particularly ET, and GPi for dystonia [35, 36]. However, choice of the DBS target in patients where there is a coexistence of tremor and dystonic features is still debated. Moreover, dystonic patients tend to be heterogeneous in terms of clinical features, while at the same time, a wide spectrum of possible aetiologies may underlie similar presentations. Eventually, clinical differences and variations in scales employed cannot be ignored when comparing different studies.

For these reasons, papers in this review were distinguished between sporadic dystonia and other clinical diagnosis with a definite cause or a precise phenotype.

In patients without a known etiology of the disease, the overall finding of our review was that thalamic surgery considerably improves tremor, but with some issues. Indeed, dystonia may be inconsistently relieved or even worsened and the benefit seems to diminish over time [4, 6, 14, 19] to the point that, in some cases, patients were implanted and switched to GPi [4]. When, conversely, a benefit was reported also on dystonic symptoms, the patients investigated had mainly cervical dystonia with dystonic tremor.

GPi-DBS showed a more limited amelioration of tremor when it was employed in patients with segmental or multifocal disease but, as expected, provided a better outcome for dystonic symptoms [6]. Head dystonic tremor seems to be more sensitive to GPi stimulation, even when unilateral [15, 17], though a complete failure was reported too [18]. Interestingly, GPi has been described also as a “rescue surgery” in cases initially treated with thalamic DBS without satisfactory control of tremor [4, 14].

So far, multi-target DBS addressing both GPi and VIM was reported in a few cases [6, 7, 9], with uniformly positive results both on dystonia and tremor. Notably, most of these procedures were staged and the second target was added after an unsatisfactory outcome with the first target.

Due to all these findings, for sporadic segmental focal or multifocal dystonia, consensus has slowly moved from a “GPi first” approach [5], which considered the nature of the disease as the key factor, to a more patient-tailored view [3, 6, 37]. Furthermore, some recent reviews have suggested that the VIM might be the best target when tremor is the most disabling feature of the disease and dystonia is absent or extremely mild, but a double-target approach should be preferred when patient characteristics are more complex or more severe dystonia is described [5, 38].

In other diseases of dystonic nature with tremor among the main clinical features, the choice of the surgical target strongly depends on the diagnosis itself. For example, task-specific tremors as PWT, whose nature is debated if being “tremulous” or “dystonic” [39], should be addressed with a thalamic approach, as showed by multiple case reports [20–23]. In myoclonic dystonia, tremor is not a usual complaint. If present, it appears to show a good response to both GPi + VIM stimulation and VIM stimulation alone [8, 27]. In fact, myoclonic dystonia has a far more complex clinical picture with a need to address myoclonus and dystonic features as well. An extensive review of the surgical treatment of this condition is beyond the aim of this article. However, a few studies have shown very good results with a dual GPi + VIM stimulation approach [40, 41].

Post-traumatic or post-stroke dystonia with tremor is another condition for which outcomes are reported, showing significant benefit both in terms of dystonic symptoms and tremor with different DBS targets.

Summarizing all these results, it may be concluded that when the disease is not sporadic, adult-onset dystonia, the diagnosis should be considered as the starting point to choose the target. The second key factor in deciding the target might be considering the severity of tremor and dystonia. Eventually, distribution of the symptoms may play a role too, though evidence about this factor is very weak.

In cases with dystonia due to a structural lesion, the target should be chosen thinking of the symptoms of the patient and the localization of the lesion responsible for the disease.

Finally, as summarized in Table 2, subthalamic area targets (STN, PSA, cZi) were reported to be effective[16, 29–34] and should be contemplated in patients with complex features or in cases where a “rescue” lead is under consideration.

### Our center experience

This retrospective case series is to our knowledge the largest one describing a four-lead approach in patients with sporadic, adult-onset dystonia, and tremor. Overall, DBS turned out to be effective in our patients, with a sustained and considerable benefit on motor symptoms (about 75% reduction in BMF-M motor subscale and a considerable reduction in tremor). Consequently, quality of life showed a definite improvement (about 50% reduction in BMF-D and a consistent benefit on quality of life, with Eq-5d increasing roughly three times after 2 years). No patients suffered permanent disability or side effects following the surgical procedure.

Our results, therefore, provide further support to the role of double targeting DBS in complex patients, which so far had only been reported in few cases [6, 7, 9]. They also confirm the feasibility, safety, and efficacy of a four-lead approach in this group of patients.

In our cohort, one patient (pt9) received VIM after GPi (implanted 2012), due to unsatisfactory tremor control. Another (pt6) received VIM + GPi because his previous VIM implant, performed in 2010 in another center, was not controlling his symptoms adequately. In all the other patients, it was decided to implant all four leads simultaneously because of their complex clinical phenotype. Indeed, since dystonia itself has been reported to worsen or to be not satisfactorily controlled after thalamic stimulation [4, 6, 7, 14, 19], there were concerns about the fact that implanting only one of the two targets would have resulted in unsatisfactory symptom control. Age was also taken in account in the decisional process: four out of nine patients were older than 65 at time of the implant. Therefore, a possible two-step approach, as suggested in literature [38] or reported [4, 6, 7, 9, 14], with the addition of a second target, if needed, was considered unlikely to be feasible.

In three patients, DBS configuration was changed from the four-lead stimulation to a VIM-only stimulation due to the onset of side effect. This change was temporary in one patient, who required GPi again, but remained unchanged in two, though a very mild deterioration of their improvement. This finding may be a consequence of the clinical context of patient revisions, where the aim has always been to optimize the stimulation for patients, balancing benefit and side effects on an individual basis.

Interestingly, a recent paper [42] has reported new-onset dystonia or marked worsening of subtle dystonic signs in patients with different tremulous disorders treated with VIM stimulation, highlighting the potential detrimental role of VIM stimulation in dystonia.

In this perspective, having four leads where stimulation can be set and changed in many more possible configurations than having GPi or VIM leads alone may help, on an individual basis, to minimize side effects without affecting the beneficial effect of DBS on symptoms.

An important point which may be worth investigating in the future is the clinical distribution of symptoms and consequently the phenotype of dystonia and tremor, and its relationship with DBS response. In fact, all three patients in our cohort who were switched from the double-target DBS to VIM stimulation alone could be classified as TAD + DT phenotype. However, one patient had significant cervical dystonia while the other two only had dystonic features confined to limbs. Interestingly, the former required GPi stimulation again at subsequent follow-up, while the latter managed to well control their symptoms with VIM stimulation only.

Finally, we found that patients with VIM + GPi DBS required relatively lower levels of VIM stimulation (lower frequency and current intensity) than patients treated with VIM-DBS only to control tremor, suggesting a synergic effect of combined VIM + GPi DBS on tremor. This could lead to a wider therapeutic window and lower risk for the onset of stimulation-induced side effect that are common when stimulating the VIM.

### Limitations of the study

Our case series has several limitations mainly related to this article being a retrospective description of nine cases, which is a relatively small sample. In addition, our patients demonstrated a moderate degree of variability in their outcomes. This fact could potentially reduce the strength of our conclusions. There are some other limitations that need to be discussed. First, the lack of a specific tremor scale performed before and after surgery. In fact, all patients were assessed using our standard of care dystonia follow-up protocol, which does not always include a precise tremor assessment. Despite all the changes in tremor were described in full in the clinical notes and letters to the patients’ General Practitioners, a more extensive tremor assessment might have provided us with further data that would have possibly helped assessing outcome and distinguishing patients according to this parameter during follow-up.

Second, we did not perform an electrophysiologic assessment of tremor which has been reported to be a supportive tool in differentiating with greater certainty tremor and other movement disorders, as myoclonus [3, 42, 43].

A further limitation is the relatively short duration of follow-up in our study, with only seven out of nine patients assessed at 24 months. A longer observation could have provided us with further information about evolution or progressive modifications in the response to DBS.

Finally, the analysis of DBS parameters compares our patients with cases either with dystonic tremor or essential tremor, thus not representing a perfectly-matched control.

## Conclusion

Our case series, in line with the published literature, supports the widely accepted use of DBS in patients with tremor and significant dystonia and provides support to the technical feasibility of multiple target implantation. Although our numbers are too small to allow us to comment on the overall risk of complications compared to two-lead case series, no significant safety issues were found.

Selection of the best target remains a subject of debate. Considering our findings and the available evidence from the literature, we believe that a tailored, patient-centreed approach based on clinical presentation may offer the best chance of good symptom control. In this scenario, GPi may be regarded as potentially not necessary in some cases of “tremor associated with Dystonia” phenotype. However, when taking in account both the potential reduction in thalamic-related side effects and the fact that a two step-approach may not be feasible in a considerable number of patients, the one step GPi + VIM may be a good option for selected patients with significant dystonia and disabling tremor.

A future study on similar patients, adopting a defined stimulation protocol, may be useful to investigate whether the combination of two targets is superior to one target or not. Furthermore, a more careful investigation of baseline differences, in terms of intensity as well as distribution of symptoms, may help for further refining target choice.

## Supplementary Information

Below is the link to the electronic supplementary material.

Supplementary file1 (DOCX 61 KB)

## Data Availability

The data that support the findings of this study are available from the corresponding author, N.P, upon reasonable request.
