# Supplementary material for: DBS in tremor with dystonia: VIM, GPi or both? A review of the literature and considerations from a single-center experience
Source: J Neurol. 2023 Jan 21;270(4):2217–29. doi: 10.1007/s00415-023-11569-6 (PMC10025201; doi:10.1007/s00415-023-11569-6)

**Supplementary Material**

**Supplementary analysis**

**Comparison between parameters of thalamic stimulation in the patients with four activated leads and a mixed cohort of Essential Tremor (ET) and dystonic patients with VIM stimulation alone**

*Subjects and Methods*

For this comparison, we included in the “4Leads” group only patients with at least one sided GPi + VIM stimulation at their last available. Additionally, due to different technologies, intensity of stimulation was measured in Volts (V) in the two patients implanted with Medtronic DBS Device and milliAmpere (mA) in the seven patients implanted with Boston Scientific. Since, impedance was not recorded at each assessment, we were not able to perform any conversion. We, therefore, included in this analysis only the seven patients whose intensity of stimulation was measured in mA.

For the control group, we used a mixed cohort of patients with ET and dystonic tremor who had been implanted with VIM only at our centre, and were on longitudinal follow-up. We also included in the control group the two patients in our cohort who did not tolerate pallidal stimulation and therefore had thalamic leads active only. Therefore, the control group consisted of 21 patients (14 male, 7 female). The mean age at surgery of this group was 62.2±8.7 years, with a disease duration at the last available appointment of 19.2±11.7 years.

For both cohort of patients, DBS parameters at their last available appointment were collected for this analysis.

Thalamic parameters included current intensity, expressed in mA, frequency of stimulation, expressed in Hz, and pulse-width expressed in μsec.

Statistical analysis was performed employing IBM-SPSS software, v 20.

Normal distribution of values was verified employing Shapiro-Wilk test.

Comparison between the two groups for each parameter was performed with T-test for independent variables or with Mann-Whitney U-test as appropriate.

*Results*

In the control group, surgery caused a mean improvement of 70.97% in Fahn-Tolosa-Marin scale in this population (pre-surgery mean value: 64.74±7.79; post-surgery mean value 19.21±11.67).

The mean value for each group for Current Intensity, Frequency and Pulse Width are represented in Supplementary Figure 1.

The intensity of thalamic stimulation in mA was normally distributed both in 4Leads and control group, according to Shapiro-Wilk test. Conversely, pulse width values and frequencies were not normally distributed in the control group. Consequently, we employed independent variables T-Test for comparing the Current, while we used MannWhitney U test for pulse width and frequency.

Statistical analysis showed that current intensity was significantly lower in the 4Leads compared with the control group (mean values 2.19±1.56 mA and 3.48±1.17 mA respectively p=0.048). The same happened for frequencies (mean values respectively 133.4±13 Hz in the 4 Leads group and 141±24.0 Hz in the control 2 Leads group, p value=0.041). No significant difference was found when comparing pulse width values (mean respectively 63±24.9 microseconds and 66±10.8; microseconds p value=0.95).

**Supplementary Table 1 - Report of DBS final settings in papers included in the review section**

*Sporadic, adult onset dystonia*

| Author & year | N of patients | Target | Left Thalamus | Right Thalamus | Left GPi | Right GPi |
| --- | --- | --- | --- | --- | --- | --- |
| *Thalamus only* | | | | | | |
| ***Vercueil 2001*** | 4 | VLp | Not reported | Not reported | - | - |
|  |  | VLp | Not reported | Not reported | - | - |
|  |  | VLp | Not reported | Not reported | - | - |
|  |  | VLp | Not reported | Not reported | - | - |
| ***Deuschl 2002*** | 1 | VIM | Not reported | Not reported | - | - |
| ***Morishita 2010*** | 2 | Right VIM | **Not activated** | **-1;+case;**  **170Hz**  **150 μs**  **3.3V** | - | - |
|  |  | VIM | **-1; +3**  **145 Hz**  **330 μs**  **2.4V** | **-2,3; +1**  **185 Hz**  **150 μs**  **4.3V** | - | - |
| ***Hedera 2013*** | 4 | Left VIM | Not reported | **Not activated** | - | - |
|  |  | Left VIM | Not reported | **Not activated** | - | - |
|  |  | Left VIM | Not reported | **Not activated** | - | - |
|  |  | VIM | Not reported | Not reported | - | - |
| ***Cury 2017*** | 3 | VIM | Not reported | Not reported | - | - |
|  |  | VIM | Not reported | Not reported | - | - |
|  |  | VIM | Not reported | Not reported | - | - |
| ***Buhmann 2013*** | 1 | VL thalamic base | **-1;+case**  **180 Hz**  **60 μs**  **1.5V** | **-9;+case**  **160 Hz**  **90 μs**  **2.2V** | - | - |
| ***Woehrle 2009*** | 1 | VIM (GPi not active) | Not reported | Not reported | - | - |
| *GPi Only* | | | | | | |
| ***Krause2004 (protocol)*** | 1 | GPi | - | - | Not reported | Not reported |
| ***Torres 2010 (protocol)*** | 1 | Gpi | - | - | Not reported | No reported |
| ***Valalik 2011*** | 1 | Pallidothomy | - | - | **Not applicable** | **Not applicable** |
| ***Hedera 2013*** | 4 | GPi | - | - | Not reported | Not reported |
|  |  |  |  |  | Not reported | Not reported |
|  |  |  |  |  | Not reported | Not reported |
|  |  |  |  |  | Not reported | Not reported |
| *GPi + Thalamus* | | | | | | |
| ***Schadt 2007*** | 1 | Bilateral Gpi + Bilateral VIM | Not reported | Not reported | Not reported | Not reported |
| ***Hedera 2013*** | 2 | Bilateral Gpi+ Left VIM | Not reported | **Not applicable** | Not reported | Not reported |
|  |  | Bilateral GPi + Left VIM | Not reported | **Previous Right thalamotomy** | Not reported | Not reported |
| ***Morishita 2010*** | 1 | Bilateral GPi + Bilateral VIM | **-2; +3**  **155 Hz**  **120 μs**  **3.1 V** | **-2,3; +case**  **60 Hz**  **180 μs**  **2.3 V** | **-3; +case**  **155 Hz**  **120 μs**  **3.2 V** | **-2,3; +case**  **60 Hz**  **180 μs**  **2.5V** |

*Other forms of dystonia*

| Author & year | N of patients | Target | Left Thalamus | Right Thalamus | Left GPi | Right GPi |
| --- | --- | --- | --- | --- | --- | --- |
| *Primary Writing Tremor* | | | | | | |
| ***Meng 2018*** | 1 | Left thalamic MrFUSS | Not applicable | - | - | - |
| ***Lyons 2013*** | 1 | Left VIM | **-3 +case**  **180 Hz**  **60 μs**  **1.5V** | - | - | - |
| ***Racette 2000*** | 1 | Left VIM | **Pattern of activation unknown**  **150 Hz**  **120 μs**  **1.5V** | **-** | - | - |
| ***Minguez castellanos***  ***1999*** | 1 | Left VIM | **-distal (1?) +case**  **130Hz**  **60 μs**  **2.2 V** | **-** | - | - |
| *Acquired, lesional dystonia* | | | | | | |
| ***Alvarez 2014*** | 1  (Post stroke) | VIM thalamotomy | **Not applicable** | **Not applicable** | - | - |
| ***Loher 2009*** | 1  (Post thrauma) | Left VL thalamotomy | **Not applicable** | **Not applicable** | **-** | **-** |
| ***Carvalho 2014*** | 1  (Post thrauma) | Right GPi | - | - | - | **-3,+case**  **185Hz**  **180 μs**  **3 V** |
| ***Woehrle 2009*** | 1  (Post thrauma) | Bilateral VIM | **Not reported** | **Not reported** | - | - |
| *Other Forms* | | | | | | |
| ***Rijntjes 2018*** | 1  Moehr-tranjenberg syndrome | Bilateral GPi | - | - | **-2,3,4; +5**  **104 Hz**  **90 μs**  **11.5 mA** | **-10,11,12; +13**  **104 Hz**  **90 μs**  **11.5 mA** |
| ***Oropilla 2010*** | 1  Myoclonic dystonia | Left VIM + Left GPi | **-2; + case**  **140 Hz**  **90 μs**  **2.1V** | - | **-2,+case**  **140 Hz**  **120 μs**  **2.3V** | - |
| ***Kuncel 2009*** | 1  Myoclonic Dystonia | Bilateral VIM | **-0,1+**  **185 Hz**  **60 μs**  **3.6 V** | **-5;+4**  **185 Hz**  **90 μs**  **3.8 V** | - | - |

**Supplementary Fig.1**

Graphic representation of parameters in patients with double target VIM and GPi stimulation (left side of figure 2A, 2B, 2C) vs patients with only thalamic VIM electrodes activated (right side of figure 2A, 2B, 2C). Fig. 2A: Current intensity expressed in mA; Fig2B: Pulse width expressed in μsc; Fig2C: frequency of stimulation expressed in Hz.


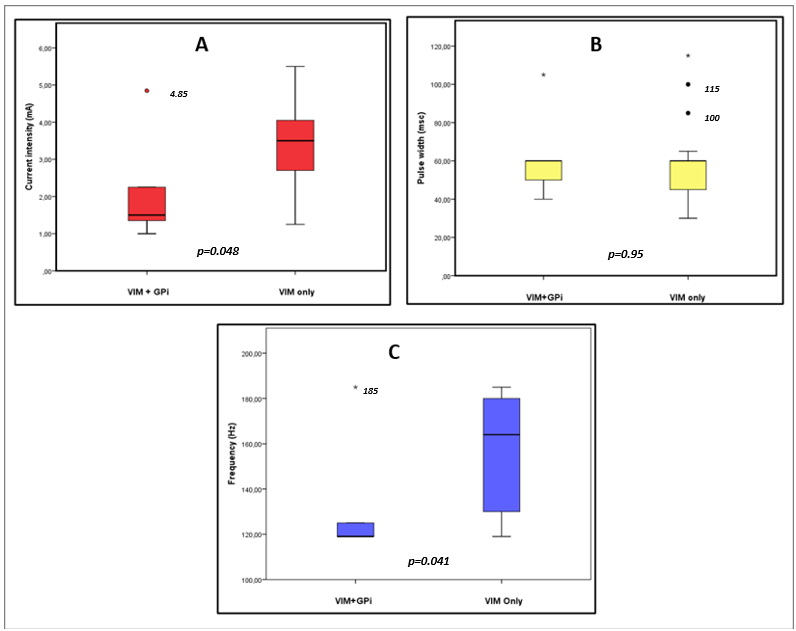

Supplement: Supplementary file 1 — Supplementary file1 (DOCX 61 KB) [file 415_2023_11569_MOESM1_ESM.docx]
